# The Intestinal Effect of Atorvastatin: *Akkermansia muciniphila* and Barrier Function

**DOI:** 10.3389/fmicb.2021.797062

**Published:** 2022-02-02

**Authors:** Tingting Cheng, Changkun Li, Linyan Shen, Shujie Wang, Xuelin Li, Chenyang Fu, Tingting Li, Bei Liu, Yanyun Gu, Weiqing Wang, Bo Feng

**Affiliations:** ^1^Department of Endocrinology, Shanghai East Hospital, Tongji University School of Medicine, Shanghai, China; ^2^National Research Centre for Endocrine and Metabolic Diseases, Ruijin Hospital, Shanghai Institute for Endocrine and Metabolic Diseases, Shanghai Jiao Tong University School of Medicine, Shanghai, China

**Keywords:** atorvastatin, NF-κB signaling pathway, gut barrier function, abnormal glucose metabolism, gut microbiota

## Abstract

Studies have shown that the cholesterol-lowering medicine statins alter the gut microbiome, induce chronic metabolic inflammation, and disrupt glycemic homeostasis. In this study, we aimed to investigate whether effects of atorvastatin (Ator) on gut microbiome and metabolic inflammation could be causally correlated. Mice at 8-week age were fed with high-fat diet (HFD) or HFD with Ator (HFD+Ator) for 16 weeks. 16S rRNA sequencing of stool and RNA sequencing of colon tissue were employed to analyze the intestinal alterations that could be induced by Ator. A human colon carcinoma cell line (Caco_2_) was used for *in vitro* experiments on barrier function. Compared to HFD, HFD+Ator induced more weight gain, impaired glucose tolerance, and led to gut microbiota dysbiosis, such as suppressing *Akkermansia muciniphila* in mice. The expressions of tight junction (TJ) proteins were attenuated in the colon, and the serum LPS-binding-protein (LBP) level was elevated in HFD+Ator mice, so as to transcriptionally activate the intestinal nuclear factor-k-gene binding (NF-κB) signaling pathway. Consistently, Ator impaired the barrier function of Caco_2_, and treatment of supernatant of *A. Muciniphila* culture could decrease the intestinal permeability and recover the attenuated expression of TJ proteins induced by Ator. In conclusion, long-term use of Ator with HFD may alter gut microbiota, induce intestinal barrier dysfunction, and hence promote chronic inflammation that contributes to disrupted glycemic homeostasis.

## Introduction

Statins, which are first-line medications, are widely prescribed for managing dyslipidemia and coronary artery disease (CAD) risks and stabilizing plaques (Koh, 2000; Colhoun et al., 2004; Almeida and Budoff, 2019; Hossaini Nasr et al., 2020). They are designed to inhibit 3-hydroxy-3-methylglutaryl-CoA (HMG-CoA) reductase, which is the rate-limiting enzyme for cholesterol synthesis with different hydrophilic properties. However, a growing number of studies based on longitude cohorts with large populations indicate that statin-use increases the risk of new-onset type 2 diabetes mellitus (T2D) in a dose-dependent manner (Rajpathak et al., 2009; Sattar et al., 2010; Preiss et al., 2011; Cederberg et al., 2015). Studies have shown that atorvastatin (Ator) as well as other statins can deteriorate glucose tolerance and promote development of diabetes *via* increasing hepatic glycogenesis (Wang et al., 2015), delaying glucose clearance (Cheng et al., 2015), inducing mitochondrial dysfunction (Urbano et al., 2017), inflammation of adipocytes (Henriksbo et al., 2019), and inhibiting adipocyte browning (Balaz et al., 2019) as well as moderately reducing β-cell functional mass *via* disrupting the mevalonate pathway to inhibit mTOR signaling (Shen et al., 2020).

Gut microbiota, as one of the most attractive fields of microbiology, medicine, and genomics in recent years, has been closely related to a variety of diseases, including atherosclerosis and T2D (Larsen et al., 2010). Studies from animal or human cohorts have found that using statins profoundly alters the gut microbiota profile. Though the featured microbiota changes induced by statins are yet to be characterized, most studies linked the altered gut microbiota composition to positive therapeutic responses of statins on CAD outcomes (Khan et al., 2018; Kim et al., 2019; Dias et al., 2020; Kummen et al., 2020; Vieira-Silva et al., 2020; Hu et al., 2021). A recent report, however, revealed the negative side of statin-induced microbiota changes, which reduced fecal butyrate and increased secondary bile acid production in a PXR-dependent manner (Caparros-Martin et al., 2017). Nevertheless, considering the role of gut microbiota in maintaining host metabolic homeostasis (Ma et al., 2019; Tilg et al., 2020; Zhao et al., 2020; Yang et al., 2021), there is a lack of evidence about how statins or statin-related microbiota alterations mediate its negative effects on glucose metabolism.

Considering the potential role of gut microbiota in regulating host glucose metabolism and known effects of statins on impairing glucose metabolism, it would be of value to study whether gut microbiota alterations induced by statins could be related to its negative effect on glycemia or the onset of T2D. Among the statins clinically prescribed, Ator, a lipophilic statin, exhibits higher efficacy in lowering serum lipids (Maxwell et al., 2017) and cardiovascular risks (Zhang X. et al., 2020). It is the earliest and one of the most widely applied kinds of statins (Athyros et al., 2010, National Institute of Diabetes and Digestive and Kidney Diseases, 2012; Adams et al., 2015). Thus, in this study, we could use Ator as a representative of statins to study how statins could compromise glucose metabolism from the perspective of gut microbiota and barrier function.

## Materials and Methods

### Experimental Animal

Eight-week-old male wild-type (WT) C57BL/6N mice were purchased from the *Model Animal Research Center of Nanjing University* (Nanjing, China), which were specific-pathogen-free (SPF) grade. The mice were divided into two groups randomly: (1) mice fed with a high-fat diet (HFD) containing 45% lipids (12451, Readydietech, China) for 4 months (HFD) and (2) mice fed with HFD containing 45% lipids and 10 mg/kg/day Ator according to our previous study (Shen et al., 2020) (HFD+Ator). All mice were housed under a 12-h light/12-h dark cycle and a 23 ± 1°C ambient temperature with free access to food and water. All procedures were approved by the Ethics Review Committee for Animal Experimentation of Tongji University.

### Metabolic Studies

Intra-peritoneal glucose tolerance test (IPGTT) was performed after 16 h of fasting, and the blood glucose of tail vein was measured as the basal blood glucose. Then, the mice were intra-peritoneally injected with glucose (2 mg/kg) and the blood glucose was measured at 15, 30, 60, and 120 min. The body composition was measured with an animal whole-body composition analyzer (100H; EchoMRI, Houston, TX, United States). Plasma total cholesterol and triglyceride were determined with an enzymatic reagent kit (Shanghai Kehua, China). Plasma insulin was determined with an ultrasensitive ALPCO insulin ELISA kit (ALPCO, Salem, NH).

### Immunofluorescence Staining

The colons were fixed with paraformaldehyde, embedded with paraffin, and sectioned with a thickness of 5 μm. Specimen sections were dewaxed and incubated with anti-Claudin1 (71-7800, Invitrogen, United States) and anti-Occludin (71-1500, Invitrogen, United States) antibodies at 4°C overnight and then incubated with Alexa Fluor conjugated antibodies (Jackson ImmunoResearch or Life Technologies, United States). Nuclei were counterstained with DAPI. Immunofluorescence images were captured using an Olympus microscope (Olympus, Tokyo, Japan) or Leica SP8 confocal microscope (Zeiss, Oberkochen, Germany).

### Real-Time Quantitative PCR

Total RNA was extracted with Total RNA Extract Kit (Promega, United States) and quantified by a micro-spectrophotometer (NanoDrop 2000, Thermo Fisher Scientific, United States). cDNA was synthesized with PrimeScript™ RT Master Mix (Takara, Japan) and then analyzed by quantitative real-time PCR with SYBR Green (Takara, Japan) on a LightCycler 480 (Applied Biosystems, United States). The relative changes in the mRNA level were calculated by the △△Ct method. The primer sequences are listed in Supplementary Table 1.

### RNA Sequencing

The total RNA of the colon was extracted with TRIzol (Invitrogen, Carlsbad, CA) and sequenced on an Illumina platform to obtain the raw data. After quality control, the raw data, namely, clean data (reads), were compared with the reference genome to obtain mapped data for subsequent transcriptional assembly and expression calculation. At the same time, the quality of the transcriptome sequencing results was evaluated, including sequencing saturation, gene coverage, the distribution of reads in different regions of the reference genome, and the distribution of reads in different chromosomes. The data were analyzed on the free online platform of *Majorbio I-Sanger Cloud Platform*.^1^

### Protein Preparation and Western Blotting

Protein was extracted with cold RIPA buffer, and the concentration was determined using a Pierce BCA Protein Assay Kit (Thermo Fisher Scientific, Waltham, MA). As for immunoblot assay, the antibodies we used were anti-Claudin1 (71-7800, Invitrogen, United States), anti-p-p65 (3033S, Cell Signaling, United States), anti-p65 (8242S, Cell Signaling, United States), and anti-Hsp90 (sc-13119, Santa Cruz Biotechnology, Dallas, TX). The target protein bands were visualized using ImageQuant LAS 4000 following the manufacturer’s guide, and Hsp90 was used as loading control to normalized band intensity. Serum lipopolysaccharide (LPS)-binding-protein (LBP) was measured with Mouse LBP ELISA Kit (Abcam, United States).

### 16S rRNA Sequencing and Sequence Analysis

Total metagenomic DNA was extracted from fecal pellets collected from 24-week-old mice just before being sacrificed using the fecal DNA isolation kit (Qiagen, United States). The concentration and purity of DNA were estimated spectrophotometrically, and the quality was assessed by agarose gel electrophoresis. The 16S rRNA V3–V4 hyper-variable region was amplified by PCR and sequenced using Illumina Miseq PE250. After quality control of the original reads, Usearch was used for data statistics and clustering analysis: amplicon sequence variants (ASVs) were obtained by 97% similarity clustering. Each ASV was considered to represent a species. Then, according to the minimum number of sequences matched to ASV, random flattening was performed and α-diversity was analyzed. A sequence of representative read was selected from each ASV and compared with the RDP database to classify each ASV and get the species abundance table for subsequent analysis. The 16S rRNA sequence was also analyzed on the free online platform of *Majorbio I-Sanger Cloud Platform* (see text footnote 1).

### *In vitro Akkermansia muciniphila* Culture

*Akkermansia muciniphila* (*Akk*) (ATCC BAA-835) was purchased from American Type Culture Collection (ATCC) and cultured in a basal mucin-based sterilized brain heart infusion broth (BHI, BD Difco) according to previous work (Becken et al., 2021) in a micro-anaerobic incubation system (Don Whitley Scientific, United Kingdom) (80% N_2_, 10% H_2_, and 10% CO_2_ at 37°C) until the optical density (λ = 600 nm) (OD_600_) reached 1.5 (Chelakkot et al., 2018). Then, the fermentation supernatants were collected and filtered with a 0.22-μm vacuum filter for subsequent use.

### *In vitro* Growth Curves of *Akkermansia muciniphila*

*Akk* was cultured in BHI medium or BHI medium containing DMSO and Ator at 37°C for 24 h in an anaerobic chamber. OD_600_ was measured at 0, 15, 24, 40, and 48 h for the bacterial growth curve.

### Cell Culture

A human colon carcinoma cell line (Caco_2_) was purchased from the cell bank of the Chinese Academy of Sciences, Shanghai, and cultured in Dulbecco’s modified Eagle medium (DMEM) supplemented with 10% fetal bovine serum, 100 IU/ml penicillin, and 100 μg/ml streptomycin in 37°C with 5% CO_2_.

### Cell Viability Assay

The cytotoxicity of Ator to Caco_2_ cells was evaluated using CCK-8 assay (Dojindo Co., Japan), measured at 450 nm with a microplate reader. In brief, cells were seeded in 96-well plates at a density of 2 × 10^4^/well and cultured for 24 h; then, cells were treated with Ator at different concentrations of 0, 5, 10, and 20 μM for 24 h, respectively. Each experiment was carried out at least five times.

### *In vitro* Trans-Epithelial Resistance Assay

Caco_2_ cells were seeded in a collagen-coated permeable polycarbonate membrane transwell chamber with 0.4-μm pores (Costar, NY, United States) placed in a six-well plate with a density of 0.2 × 10^6^/well and were grown as monolayers for about 7–9 days. The trans-epithelial resistance (TEER) values of all monolayers were measured with a Millicell ERS voltohmmeter (Millipore, United States) before and after the treatment. When the TEER no less than 500 Ω⋅cm^2^, DMSO (1:1,000, v/v), 10 μM of Ator, and fermentation supernatants of *Akk* (1:1,000) were added, respectively, to the basal and apical chambers of transwell supports. Then, the paracellular permeability was monitored for the next 4 days.

### Statistical Analysis

The data were analyzed with SPSS 23.0 (SPSS Inc., Chicago, IL), and the plots were generated using Prism 8 (GraphPad software). Student’s two-tailed *t*-test, one-way ANOVA, and ANCOVA were used to determine statistical differences. Spearman correlation, Pearson correlation, and simple linear regression analysis were used to determine the relationship of genes and protein levels with the differential microbiota. All results were expressed as mean ± SEM, and statistical significance was assigned to *P* < 0.05.

## Results

### Atorvastatin Induced Abnormal Glycemia in High-Fat Diet Mice

After 16-week treatment of Ator with HFD, the metabolic phenotypes of these mice were consistent with previous reports (Wang et al., 2015; Shen et al., 2020). HFD+Ator mice showed significantly increased body weight compared to the HFD mice (Supplementary Figure 1A, *P* < 0.05), lowered serum total cholesterol (TC) (Supplementary Figure 1B, *P* < 0.01), and unaltered serum total triglyceride (TG) levels (Supplementary Figure 1C, *P* = 0.24). Ator did not alter the percentage of epididymal fat weight (Supplementary Figure 1D, *P* = 0.77) but impaired glucose tolerance (Supplementary Figures 1E,F, *P* < 0.05). Hence, in this study, we successfully established the long-term use of Ator-induced hyperglycemia mouse model.

**FIGURE 1:**
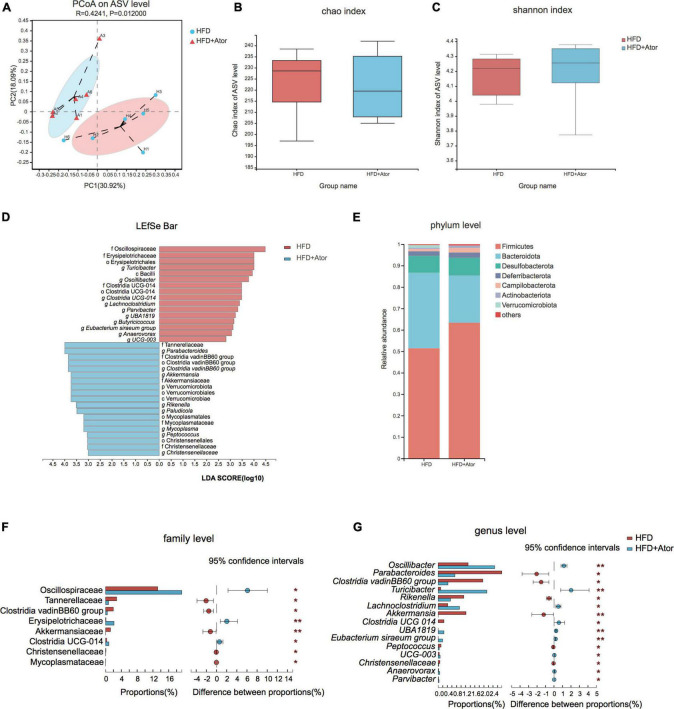
Ator induced gut microbe alteration in mice. **(A)** Principal co-ordinates analysis (PCoA) of gut microbe of HFD and HFD+Ator mice at ASV level. **(B)** Chao index for microbiota enrichment. **(C)** Shannon index for microbiota diversity. **(D)** Linear discriminant analysis (LDA) of altered gut microbiota composition associated with Ator treatment. **(E)** Taxonomy composition alteration at the phylum level in each group. **(F,G)** Bar plots showing the differential ASVs at family level **(F)** and genus level **(G)**. *P < 0.05, **P < 0.01, *n* = 6, Wilcoxon rank-sum test.

### Long-Term Use of Atorvastatin Altered the Gut Microbe Composition in High-Fat Diet Mice

Next, we sought to compare the gut microbiota compositions of stool between HFD-fed mice treated with Ator and vehicle by 16S rRNA sequencing. A total of 273,865 sequences were generated from 12 samples. An average of 22,822 ± 2117 sequences were recovered per sample and used for comparative analyses. The number of ASVs reached saturation in rarefaction analysis (Supplementary Figure 2A), suggesting that the amount of sequencing data was sufficient to reflect the vast majority of microbial diversity information. Principal co-ordinates analysis (PCoA) illustrated that the gut microbiota compositions were distinct between HFD mice and HFD+Ator mice (Figure 1A). However, profile analysis showed no difference in bacterial taxonomy diversity or richness between the two groups (Figures 1B,C). In addition, by differential analysis for relative abundance (RA), we observed that several ASVs had their RAs altered by Ator treatment (Figure 1D and Supplementary Figure 2B). At the phylum level, Ator induced a decrease in the Bacteroidetes/Firmicutes ratio (Figure 1E). At the family level, long-term use of Ator increased Oscillospiraceae and Erysipelotrichaceae and lowered Tannerellaceae, Clostridia vadinBB60 group, and Akkermansiaceae (Figure 1F and Supplementary Table 2). At the genus level, stools from HFD+Ator mice were enriched with *Oscillibacter*, *Turicibacter*, *Anaerovorax*, and *Parvibacter* but depleted with *Parabacteroides*, *Akkermansia*, *Rikenella*, and *Christensensellaces* (Figure 1G and Supplementary Table 2).

### Atorvastatin Transcriptionally Induced Inflammation in Mice Intestine

**FIGURE 2:**
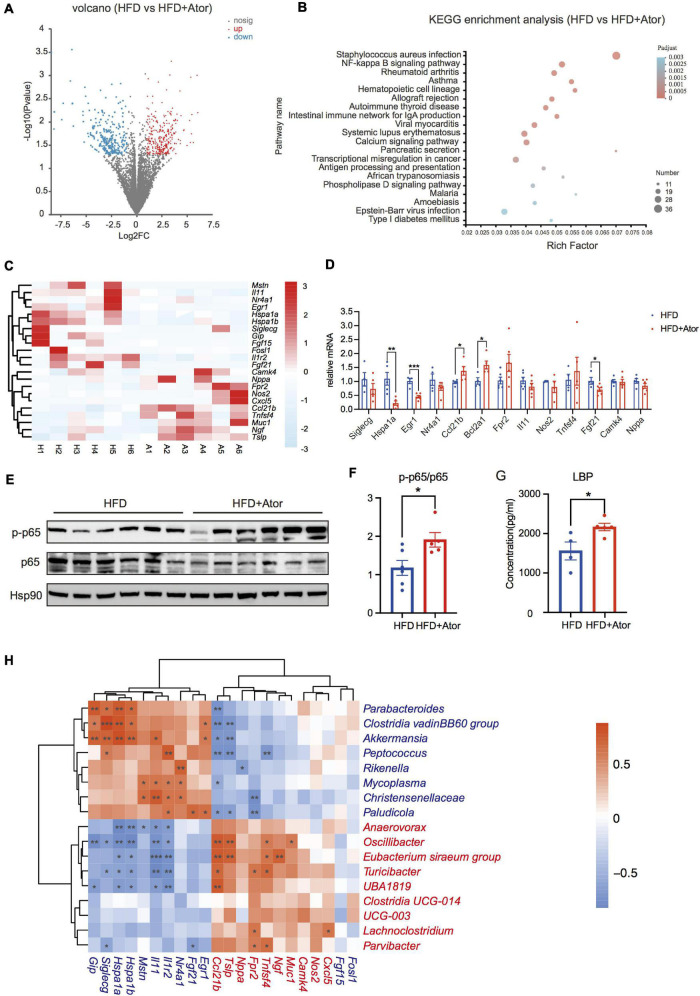
Ator induced inflammation of colon. **(A)** A volcano map of RNA sequencing from mouse colon tissue, *n* = 6. **(B)** The KEGG pathway enrichment analysis of colon RNA Sequencing between the two groups. **(C)** A heat map showing the NF-κB pathway related genes with different expression from colon RNA sequencing, *n* = 6. **(D)** RT-PCR of NF-κB pathway-related genes in colon, *n* = 4–6. **(E)** Western blot of p65 and p-p65 in colon, *n* = 5–6. **(F)** Densitometric analysis of Western blot bands in **(E)**, *n* = 4–6. **(G)** Serum LBP levels of mice fed with HFD or HFD+Ator for 16 weeks, *n* = 4–5. **(H)** Spearman correlation analysis of differentially expressed NF-κB signaling pathway-related genes with the differential ASVs at genus level, *n* = 6. The IDs of different expressive genes and differential ASVs features are highlighted in red (Ator-positive) and blue (Ator-negative). Data are presented as mean ± SEM. *P < 0.05, **P < 0.01, ***P < 0.001, unpaired two-tailed Student’s *t*-test.

To further elucidate the effect of Ator on the gut, we performed RNA sequencing to study the transcriptome profile of colon epithelia. A total of 761 differentially expressed genes were found between HFD mice and HFD+Ator mice, and 361 genes were downregulated, while 400 genes were upregulated in HFD+Ator mice (Figure 2A, Supplementary Figure 3A, and Supplementary Table 3). KEGG pathway enrichment analysis found enrichment of the nuclear factor-k-gene binding (NF-κB) signaling pathway in the colon of HFD+Ator mice (Figure 2B). We confirmed some of the RNAseq data with real-time quantitative PCR (RT-PCR) that Ator treatment elevated expressions of NF-κB downstream genes like *Bcl2a1*, *Ccl21b*, and NF-κB upstream activating factors like *Fpr2*, *Muc1*, and *Tnfsf4*. Meanwhile, the expression of anti-inflammation genes, such as *Hspa1a*, *Egr1*, *Il1r2*, and *Il11*, the negative NF-κB upstream regulators, was confirmed downregulated in HFD+Ator mice (Figures 2C,D). Moreover, the colon p65 phosphorylation level, the key marker of activation of the NF-κB signaling pathway (Oeckinghaus et al., 2011), was upregulated in HFD+Ator mice (Figures 2E,F). Therefore, we proposed that long-term use of Ator may transcriptionally activate the NF-κB signaling pathway that could lead to intestinal chronic inflammation.

However, in *in vitro* experiments, Ator treatment could not directly activate the NF-κB signaling pathway in the Caco_2_ cell line (Supplementary Figures 3D,E), suggesting that the intestinal inflammation after Ator treatment *in vivo* could be indirect, and we thought it might be derived from its regulations on gut microbiota. Paralleled with this assumption, the serum level of LBP, which is a proxy for LPS in circulation, was also elevated in HFD+Ator mice compared to that in HFD mice (Figure 2G). The Ator-depleted taxa, such as *Akkermansia*, *Clostridia vadinBB60 group*, and *Parabacteroides*, were negatively associated with the transcription of pro-inflammatory genes, while Ator-positive taxa *Turicibacter* and *Oscillibacter* were positively associated with pro-inflammatory genes (Figure 2H). In all, our results suggested that Ator induced the activation of the intestinal NF-κB signaling pathway and chronic inflammation in the gut that may be associated with the gut microbiome alteration.

### Atorvastatin Impaired the Gut Barrier Function

**FIGURE 3:**
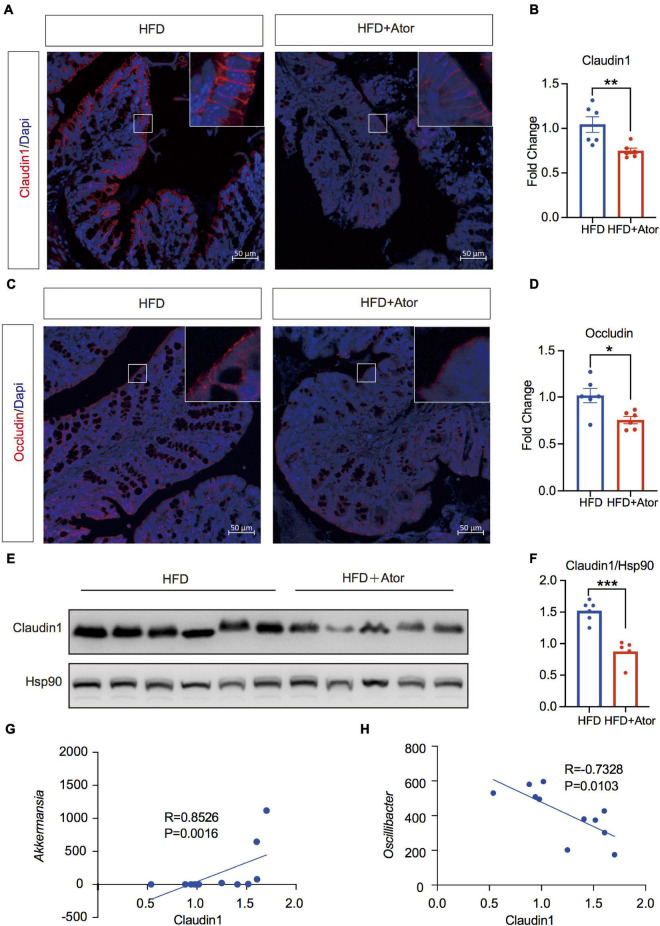
Ator impaired barrier function in colon associated with gut microbiota. **(A,B)** Representative images of immunofluorescence staining of Claudin1 in colon and the quantitative analysis of fluorescence intensity, *n* = 6. **(C,D)** Representative images of immunofluorescence staining of Occludin in colon and the quantitative analysis of fluorescence intensity, *n* = 6. **(E,F)** Western blot of Claudin1 in colon and the quantitative analysis of grayscale value, *n* = 5–6. **(G)** Spearman correlation analysis of Claudin1 protein level with *Akk*, *n* = 6. **(H)** Pearson correlation analysis of Claudin1 protein level with *Oscillibacter*, *n* = 6. For immunofluorescence staining, we used three sections for each mouse; for quantitative analysis of fluorescence intensity, we use five views for each section. Original magnification × 200. Data are presented as mean ± SEM. *P < 0.05, **P < 0.01, ***P < 0.001, unpaired two-tailed Student’s *t*-test.

**FIGURE 4:**
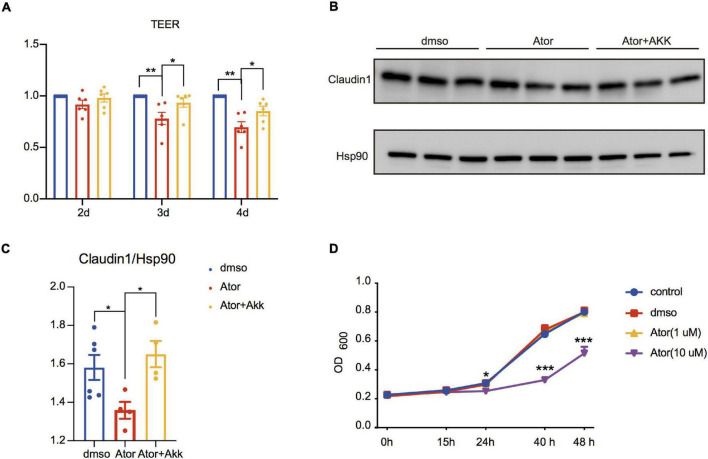
*Akk* fermentation supernatants restored Ator-induced gut barrier dysfunction in Caco_2_ cell. **(A)** Ratios of TEER value in different treatments compared to DMSO were analyzed on the second, third, and fourth days after Ator and *Akk* supernatant treatment, *n* = 6. **(B)** Western blot of Claudin1 on the fourth day after Ator and *Akk* supernatant treatment in Caco_2_, *n* = 4–6. **(C)** Densitometric analysis of bands in Western blot in **(B)**, *n* = 4–6. **(D)** Growth curve of *Akk* treated with Ator with different concentrations, *n* = 3. Data are presented as mean ± SEM. *P < 0.05, **P < 0.01, ***P < 0.001, one-way ANOVA for three groups.

Elevated chronic inflammation is commonly attributed to a disrupted intestinal barrier and enhanced bacterial LPS production (Zhang X. Y. et al., 2020). The transcriptional changes of tight junction (TJ) proteins were not suggested by Ator in RNAseq data. However, *in situ* immunolabeling of Claudin1 and Occludin, the two key components of TJ, showed significantly lowered fluorescence intensity in HFD+Ator mice gut epithelia compared to HFD mice (Figures 3A–D). Western blotting of colon tissue also showed decreased levels of Claudin1 (Figures 3E,F). In order to determine whether the attenuated TJ protein expression was related to the altered microbiota, we performed Spearman correlation between the colon protein level of Claudin1 that was determined by densitometric analysis of bands from Western blotting and the RA of Ator-induced altered microbiota at the genus level. We found that *Akkermansia*, *Parabacteroides*, and *Clostridia vadinBB60 group* showed positive association (Figure 3G and Supplementary Figures 4A,B), while *Oscillibacter* and *Turicibacter* showed negative association (Figure 3H and Supplementary Figure 4C) with Claudin1 levels. We next tested the effect of Ator on barrier function *in vitro* in the Caco_2_ cell line. Ator began to decrease the TEER from the second day it was applied in the culture media (Figure 4A). In addition, results of Western blotting also showed downregulation of Claudin1 levels by Ator treatment (Figures 4B,C). Hence, we suggest that the negative effect of long-term use of Ator on the gut barrier function was associated with direct downregulation of TJ proteins in addition to the alteration of gut microbiome.

### *Akkermansia muciniphila* Rescued the Effect of Atorvastatin on the Gut Barrier

*Akk* has been known as a next-generation probiotic for its treatment effect on metabolic diseases (Dao et al., 2016; Plovier et al., 2017; Depommier et al., 2019; Macchione et al., 2019), and one of the underlying mechanisms is to improve intestinal barrier integrity and ameliorate the chronic inflammation and insulin resistance (Lukovac et al., 2014; Chelakkot et al., 2018; Brandsma et al., 2019). The negative effect of Ator on the RA of *A. munciniphila* shown in Figure 1 prompted us to consider whether *Akk* could also mediate the effect of Ator on the barrier function. We treated Caco_2_ simultaneously with Ator and the supernatant of a strain of *Akk* (ATCC BAA-835). We found that compared to the vehicle, Ator treatment alone significantly reduced the TEER (Figure 4A) and the protein levels of Claudin1 (Figures 4B,C) in Caco_2_, which was notably restored by supplement of the *Akk* fermentation supernatants. To further confirm whether Ator exerted inhibit effect on the commensal *Akk*, we then performed the *in vitro* growth experiment on *Akk* with treatment of Ator. The result showed that Ator at a concentration of 10 μM significantly suppressed the growth of *Akk* (Figure 4D). Thus, we thought that long-term use of Ator might directly compromise the intestinal barrier function that was partially mediated by its effect *via* inhibiting *Akk*. *Akk* could serve as a probiotic taxon to prevent the detrimental effect of statins on the gut barrier function.

## Discussion

**FIGURE 5:**
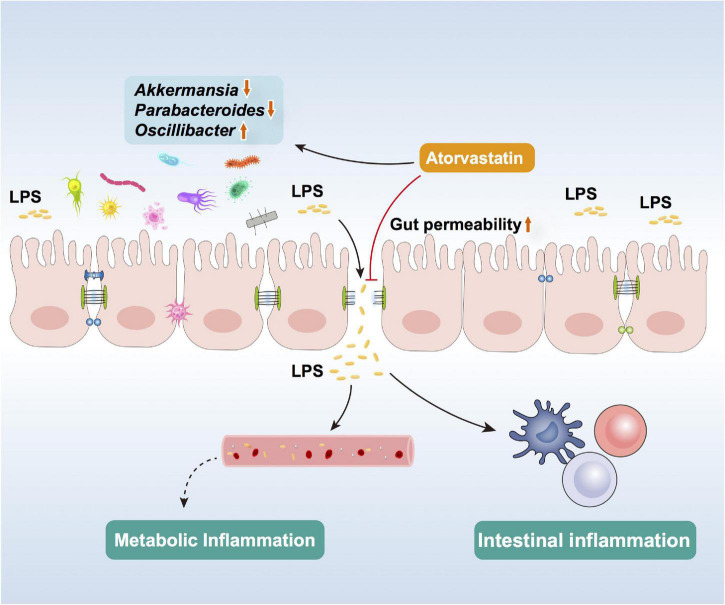
Schematic of Ator effects in altering gut microbiota, impairing barrier function, and inducing colon inflammation.

In the present study, 16-week treatment of Ator increased body weight and impaired glucose tolerance in HFD mice. We found that Ator disturbed gut microbiome symbiosis that was characterized by increasing *Oscillibacter* and decreasing *Akkermansia*. The transcriptional activation of the NF-κB signaling pathway and decreased protein level of TJ proteins in the colonic epithelium in HFD+Ator mice showed the potential of Ator to induce gut microbiota alteration, promote inflammation, and disrupt the barrier function. The *in vitro* experiment using Caco_2_ cell line further confirmed the direct negative effect of Ator on the barrier function and TJ proteins, which could be rescued by supplementing with the supernatant of a strain of *Akk* (ATCC BAA-835). In summary, our results suggest that the abnormal glycemia after long-term Ator treatment might be partly mediated by its effect on gut including altering the gut microbiota and impairing the intestinal barrier function to promote chronic inflammation. Gut microbiota could serve as a potential therapy target for this side effect of statins (Figure 5).

In recent years, the important role of gut in the pathogenesis and therapy of T2D is widely recognized and heavily investigated (Larsen et al., 2010; Tilg and Moschen, 2014; Tilg et al., 2020; Yang et al., 2021). Among multiple potential mechanisms that have been found, the increased intestinal permeability to induce metabolic endotoxemia and insulin resistance has been widely recognized and accepted (Cani et al., 2007, 2008; Tilg et al., 2020; Zhao et al., 2020; Yang et al., 2021). Increased intestinal permeability manifests impaired intestine barrier function, which comprises mainly mechanical (mucus, TJ, epithelial layer), humoral (defensins, IgA), and immunological elements (lymphocytes, innate immune cells) and which constitutes the interface of gut microbes and host cells. Both key TJ proteins Occludin and Claudin1 (Lee, 2015; Blander, 2016) were inhibited by Ator, indicating that long-term treatment of Ator with HFD could directly intervene in the normal barrier function. The mechanism underlying the Ator regulation on TJ proteins requires further investigation; it seemed not to be at the transcriptional level, as suggested by our data. Of note, KEGG pathway enrichment analysis demonstrated that the genes regulating IgA production and antigen processing and presentation were also altered by Ator, suggesting that the immune barrier of gut could also be affected by Ator treatment, which awaits further investigation.

Consistent with the impaired gut barrier function, we found serum LBP was elevated and the colon NF-κB signaling pathway was transcriptionally activated by Ator treatment, indicating the induction of chronic inflammation. The transcriptome of pancreatic islets from mice with similar Ator treatment has also shown activated NF-κB signaling and enhanced metabolic inflammation (Shen et al., 2020). Though the anti-inflammatory effects of statins have been reported in traumatic brain injury, HFD-induced renal injury, endothelial dysfunction, and cardiac and adipose tissue (Golia et al., 2014; Xu et al., 2017; Yamada et al., 2017; da Silva et al., 2018; Pengrattanachot et al., 2020), it has been reported that statins can induce insulin resistance *via* activation of caspase-1/IL-1β inflammasomes in adipose tissue (Henriksbo et al., 2019) and Ator could trigger liver toxicity in diabetic rats by promoting the generation of inflammation (Zeng and Liu, 2019). Moreover, we did not find that Ator could directly alter the NF-κB signaling pathway in Caco_2_ cells. Thus, we proposed that long-term treatment of Ator with HFD could cause “leaky gut” and elevate blood LPS that contribute to the chronic low-level metabolic inflammation and could underlie the diabetogenic effect of Ator.

There are several studies on animal or cohorts observing that statins can alter the gut microbiota profile (Dias et al., 2020; Kummen et al., 2020; Hu et al., 2021), albeit without unified conclusion on the featured gut microbiome induced by statins. Of note, a study has reported that statins reduce butyrate production (Caparros-Martin et al., 2017), implying a a potential to decrease intestinal barrier function (Chen et al., 2018). In our study, the main gut microbiota changes induced by Ator treatment were similar to those in metabolic diseases such as diabetes and obesity, which have potential to impact the gut barrier function or promote metabolic inflammation (Olson et al., 2018; Wang et al., 2019; Reitmeier et al., 2020), including decreased *Akkermansia*, *Parabacteroides*, and *Rikenella*, and the flourish of LPS producers like *Oscillibacter*. Commensal *Akk*, though fed on mucin, the mechanical gut barrier (Arumugam et al., 2011), can otherwise fortify the barrier *via* secretion of SCFA (Lukovac et al., 2014; Brandsma et al., 2019) and extracellular vesicles (AmEVs) (Chelakkot et al., 2018). Our study observed that *Akk* was suppressed in HFD+Ator mice and in *in vitro* culture treated with Ator. Moreover, the culture supernatant of *Akk* could rescue the impaired barrier function of Caco_2_ induced by Ator treatment. Hence, we thought that *Akk* might serve as an important target of Ator in gut microbiota to mediate its negative effect on the gut barrier and chronic metabolism inflammation.

There are some limitations in our study. First, though we thought that the deficient barrier function in the colon by Ator treatment might cause elevated LBP and activation of the NF-κB signaling pathway, the possibility that the increased LPS produced by the gut microbiota could also contribute to gut barrier function disruption could not be excluded. Second, our experiments did not include other strains of *Akk*, particularly the one from the experiment mice. Third, we did not test the potential risks of probiotics (Doron and Snydman, 2015), including systemic infection, excessive immune stimulation, or gene transfer (Drago, 2019). Future studies on multiple strains of *Akk* are unquestionably required to sufficiently evaluate efficacy and safety before employing *Akk* as a probiotic from bench to bed.

## Conclusion

In conclusion, our study suggested that Ator may alter the gut microbiota symbiosis and induce dysfunction of the intestinal barrier that might promote chronic metabolic inflammation and partly mediate the diabetogenic effect of Ator. The commensal *Akk* residing in the gut might serve as a potential target to prevent or treat statin-induced hyperglycemia.

## Data Availability Statement

The datasets presented in this study can be found in online repositories. The data presented in the study are deposited in the BioProject repository, accession numbers are PRJNA771633 and PRJNA770450.

## Ethics Statement

The animal study was reviewed and approved by the Ethics Review Committee for Animal Experimentation of Tongji University.

## Author Contributions

YG, CL, LS, WW, and BF designed the research and revised the manuscript. TC and LS performed the animal experiments. TC, YG, and BF wrote the draft of manuscript. TC, CL, LS, TL, YG, XL, WW, and BF participated in discussion. TC and TL performed the cell experiments and molecular biology experiments. CL performed the microbiology-related experiments. TC, SW, BL, and CF analyzed the data. BF, WW, and YG supervised the study and had full access to all the data in the study and take responsibility for the integrity of the data and the accuracy of the data analysis. All authors contributed to the article and approved the submitted version.

## Conflict of Interest

The authors declare that the research was conducted in the absence of any commercial or financial relationships that could be construed as a potential conflict of interest.

## Publisher’s Note

All claims expressed in this article are solely those of the authors and do not necessarily represent those of their affiliated organizations, or those of the publisher, the editors and the reviewers. Any product that may be evaluated in this article, or claim that may be made by its manufacturer, is not guaranteed or endorsed by the publisher.

## Footnotes

1
https://cloud.majorbio.com/
