# Supplementary material for: The Intestinal Effect of Atorvastatin: Akkermansia muciniphila and Barrier Function
Source: Front Microbiol. 2022 Feb 2;12:797062. doi: 10.3389/fmicb.2021.797062 (PMC8847773; doi:10.3389/fmicb.2021.797062)
Supplement: Supplementary file 1 [file Data_Sheet_1.docx]

Supplementary Material

**Supplementary Tables**

**Supplementary Table 1** Primer list for qRT-PCR

| Genes | 5'‐Forward Primer‐3' | 5'‐Reverse Primer‐3' |
| --- | --- | --- |
| GAPDH | CATGTTCCAGTATGACTCCACTC | GGCCTCACCCCATTTGATGT |
| Hspa1a | TGGTGCAGTCCGACATGAAG | GCTGAGAGTCGTTGAAGTAGGC |
| Egr1 | TCGGCTCCTTTCCTCACTCA | CTCATAGGGTTGTTCGCTCGG |
| Nr4a1 | TTGAGTTCGGCAAGCCTACC | GTGTACCCGTCCATGAAGGTG |
| Ccl21b | TCCCTACAGTATTGTCCGAGGC | ATCAGGTTCTGCACCCAGCCTT |
| Bcl2a1 | GGCTGAGCACTACCTTCAGTA | TCCCTACAGTATTGTCCGAGGC |
| Fpr2 | GCCTTTTGGCTGGTTCCTGTGT | CAAATGCAGCGGTCCAAGGCAA |
| Il11 | TGTTCTCCTAACCCGATCCCT | CAGGAAGCTGCAAAGATCCCA |
| Nos2 | GAGACAGGGAAGTCTGAAGCAC | CCAGCAGTAGTTGCTCCTCTTC |
| Tnfsf4 | GGAAGAAGACGCTAAGGCTGGT | CTGGTAACTGCTCCTCTGAGTC |
| Fgf21 | CTGCTGGGGGTCTACCAAG | CTGCGCCTACCACTGTTCC |
| Camk4 | GAGAACCTCGTCCCGGATTAC | ACACAATGGATGTAGCACCCC |
| Nppa | GCTTCCAGGCCATATTGGAG | GGGGGCATGACCTCATCTT |
| Siglecg | GTCCCAGACTTGCATGAGAATC | GACCCAGCTCAGTGTAGCA |

**Supplementary Table 2** Relative abundance of the differential microorganisms between the two groups.

**Supplementary Table 3** Genes with differential expression in colon (RNAseq) between the two groups.

## Supplementary Figures

**Supplementary Figure 1.** Metabolic phenotypes of mice fed with HFD or HFD+Ator for 16 weeks. **(A)** Body weight, n=6, unpaired two-tailed Student *t* test. **(B)** Serum cholesterol levels, n=5-6, Analysis of Covariance (ANCOVA) with body weight as the covariate. **(C)** Serum triglyceride levels, n=5-6, ANCOVA with body weight as the covariate. **(D)** The percentage of epididymal fat weight, n=6, unpaired two-tailed Student *t* test. **(E-F)** Intraperitoneal glucose tolerance test (IPGTT) and the average area under the curve, n=5-6, unpaired two-tailed Student *t* test. Data are presented as the mean±SEM. *P<0.05, ** P<0.01.


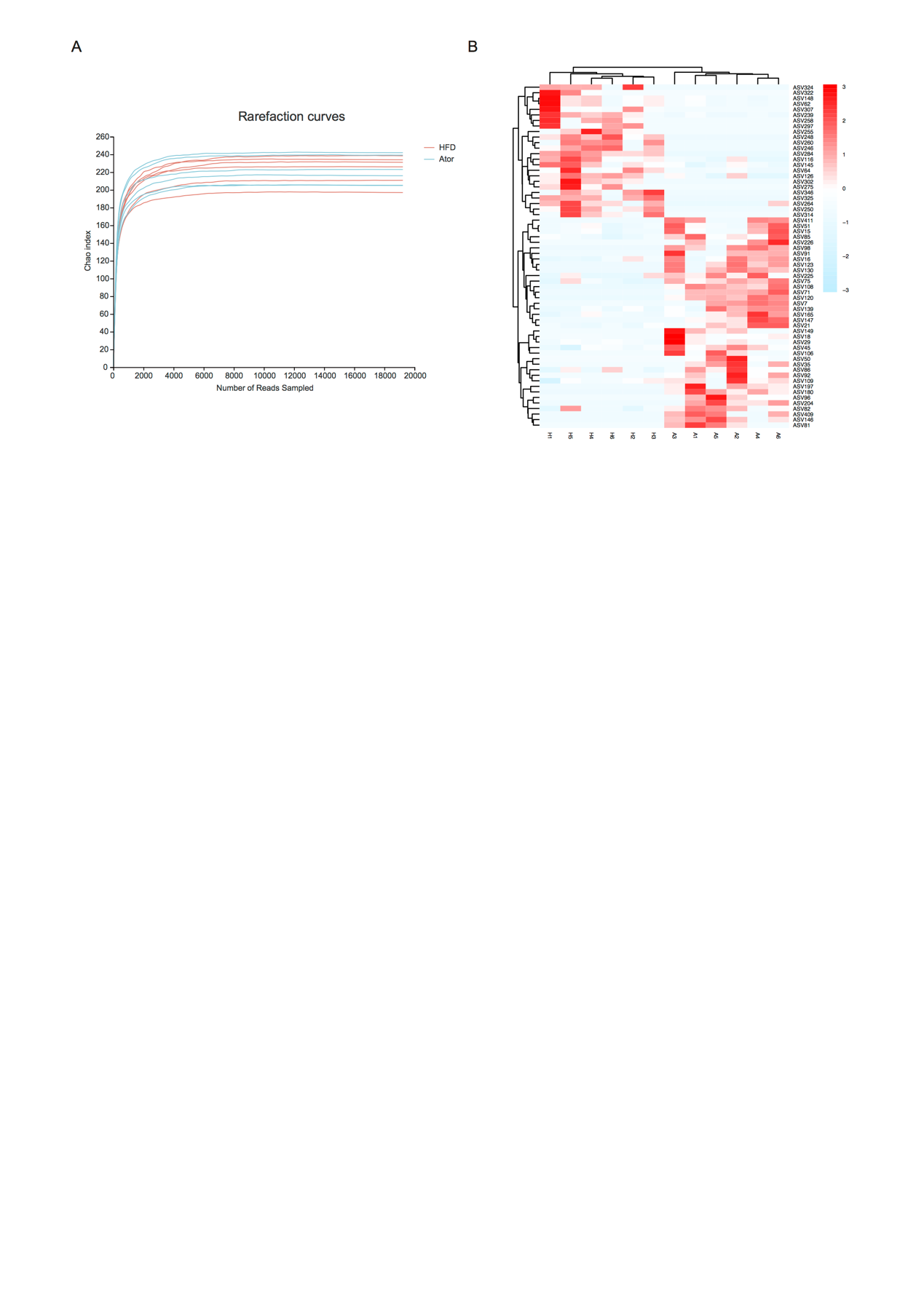


**Supplementary Figure 2.** Profile analysis of 16s rRNA sequencing of gut flora in mice fed with HFD or HFD+Ator. **(A)** The rarefaction curve of 16s rRNA sequencing which reflects the microbial diversity of each sample in different sequencing quantity. **(B)** A heat-map demonstrating differential ASVs between HFD and HFD+Ator.


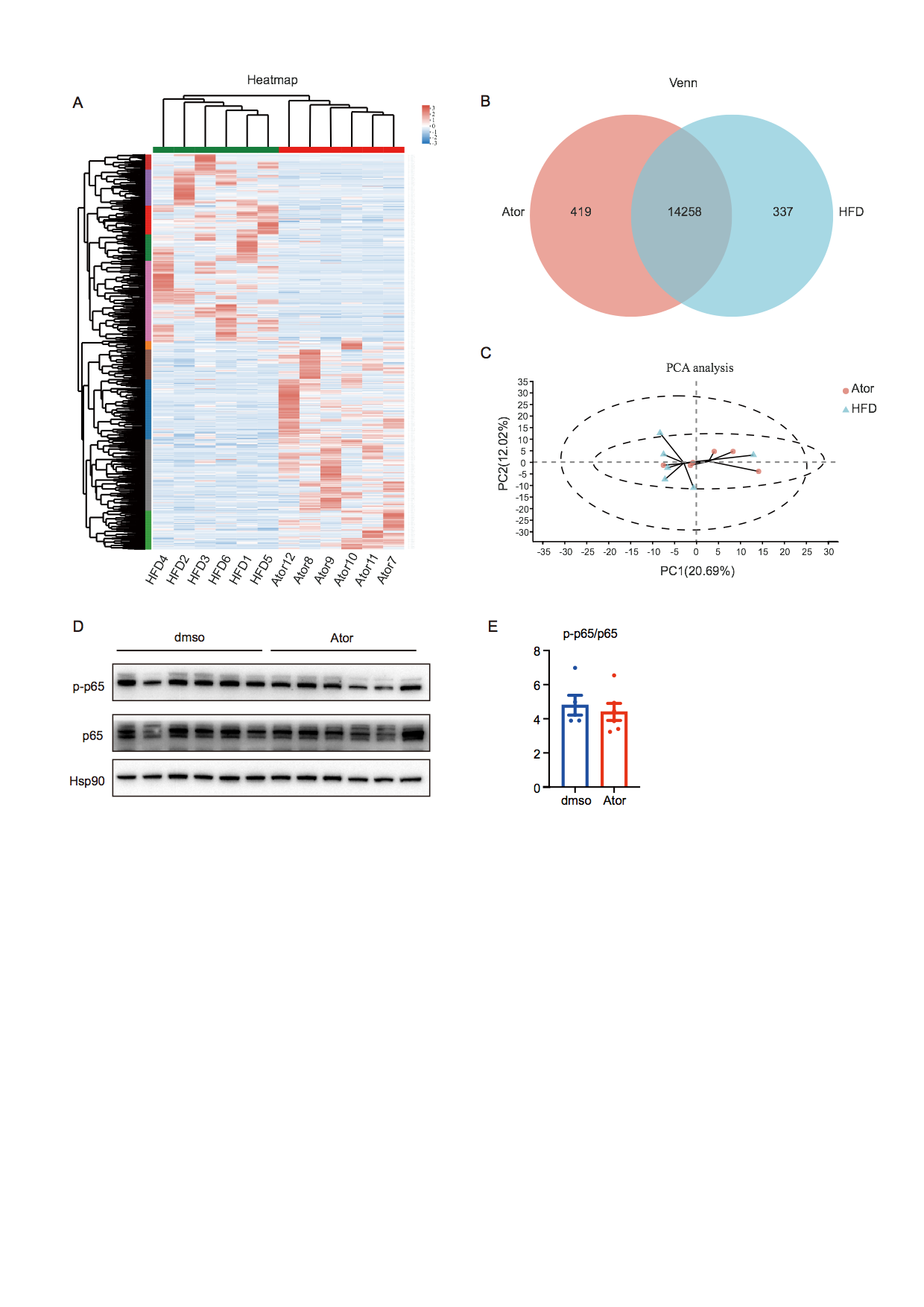


**Supplementary Figure 3.** RNA sequencing of colon. **(A)** A heat-map of the profile of differentiated expressed genes in colon from mice fed with HFD and HFD+Ator for 16 weeks. **(B)** Venn diagram showed the number of genes differentiated expressed in colon tissue between HFD and HFD+Ator (a threshold of P<0.05 or FDR≤0.2 and fold change≥2). **(C)** Principal component analysis (PCA) of gene expression between the two groups, n=6. **(D)** Western blot of p65 and p-p65 in Caco2 cell line, n=6. **(E)** Densiometric analysis of western bands in D, n=6. Data are presented as the mean±SEM.

**Supplementary Figure 4.** Spearman correlation analysis of Claudin1 protein level with the differential ASVs at genus level: *Clostridia vadinBB60 group* **(A)**; *Parabacteroides* **(B)**; *Turicibacter* **(C)**, n=6.
